# Organizational readiness to change assessment (ORCA): Development of an instrument based on the Promoting Action on Research in Health Services (PARIHS) framework

**DOI:** 10.1186/1748-5908-4-38

**Published:** 2009-07-14

**Authors:** Christian D Helfrich, Yu-Fang Li, Nancy D Sharp, Anne E Sales

**Affiliations:** 1 Northwest HSR&D Center of Excellence, VA Puget Sound Healthcare System, Seattle, Washington, USA; 2 Department of Health Services, University of Washington School of Public Health, Seattle, Washington, USA; 3 Department of Biobehavioral Nursing and Health Systems, University of Washington, School of Nursing, Seattle, Washington, USA; 4 Faculty of Nursing, University of Alberta, Edmonton, Alberta, Canada

## Abstract

**Background:**

The Promoting Action on Research Implementation in Health Services, or PARIHS, framework is a theoretical framework widely promoted as a guide to implement evidence-based clinical practices. However, it has as yet no pool of validated measurement instruments that operationalize the constructs defined in the framework. The present article introduces an Organizational Readiness to Change Assessment instrument (ORCA), organized according to the core elements and sub-elements of the PARIHS framework, and reports on initial validation.

**Methods:**

We conducted scale reliability and factor analyses on cross-sectional, secondary data from three quality improvement projects (n = 80) conducted in the Veterans Health Administration. In each project, identical 77-item ORCA instruments were administered to one or more staff from each facility involved in quality improvement projects. Items were organized into 19 subscales and three primary scales corresponding to the core elements of the PARIHS framework: (1) Strength and extent of evidence for the clinical practice changes represented by the QI program, assessed with four subscales, (2) Quality of the organizational context for the QI program, assessed with six subscales, and (3) Capacity for internal facilitation of the QI program, assessed with nine subscales.

**Results:**

Cronbach's alpha for scale reliability were 0.74, 0.85 and 0.95 for the evidence, context and facilitation scales, respectively. The evidence scale and its three constituent subscales failed to meet the conventional threshold of 0.80 for reliability, and three individual items were eliminated from evidence subscales following reliability testing. In exploratory factor analysis, three factors were retained. Seven of the nine facilitation subscales loaded onto the first factor; five of the six context subscales loaded onto the second factor; and the three evidence subscales loaded on the third factor. Two subscales failed to load significantly on any factor. One measured resources in general (from the context scale), and one clinical champion role (from the facilitation scale).

**Conclusion:**

We find general support for the reliability and factor structure of the ORCA. However, there was poor reliability among measures of evidence, and factor analysis results for measures of general resources and clinical champion role did not conform to the PARIHS framework. Additional validation is needed, including criterion validation.

## Introduction

The Promoting Action on Research Implementation in Health Services, or PARIHS, framework is a theoretical framework widely promoted as a guide to implementation of evidence-based clinical practices [1-5]. It has been the subject of much interest and reference by implementation researchers [6-13], at a time when theoretical frameworks are needed to guide quality improvement activities and research [14-16].

However, a key challenge facing the PARIHS framework is that it has as yet no pool of validated measurement instruments that operationalize the constructs defined in the framework, and the PARIHS framers have prioritized development of diagnostic or evaluation tools [5]. Currently the only published instruments related to PARIHS are a survey on clinical practice guideline implementation [13], and the Context Assessment Index (CAI) [17], both of which have important limitations for assessing readiness to implement a specific evidence-based practice.

The purpose of the present article is to introduce an organizational readiness to change assessment instrument (ORCA), derived from a summative evaluation of a quality improvement study and organized in terms of the PARIHS framework, and to report scale reliability and factor structures. The ORCA was developed by the Veterans Health Administration (VHA) Quality Enhancement Research Initiative for Ischemic Heart Disease and was initially field tested in three quality improvement projects and studies. The scales were designed to assess organizational readiness to change in preparation for testing interventions designed to implement evidence-based changes in clinical practice. The scales are intended for diagnostic use, to identify needs or conditions that can be targeted by implementation activities or resources, and to provide a prognosis of the success of the change effort at the organizational level.

## Background

### The PARIHS framework

The PARIHS framework was developed to represent essential determinants of successful implementation of research into clinical practice [1]. The PARIHS framework posits three core elements that determine the success of research implementation: (1) Evidence: the strength and nature of the evidence as perceived by multiple stakeholders; (2) Context: the quality of the context or environment in which the research is implemented, and (3) Facilitation: processes by which implementation is facilitated. Each of the three core elements, in turn, comprises multiple, distinct components.

**Evidence **includes four components, corresponding to different sources of evidence: (1) research evidence from published sources, or participation in formal experiments, (2) evidence from clinical experience or professional knowledge, (3) evidence from patient preferences or based on patient experiences, including those of caregivers and family; and (4) routine information derived from local practice context, which differs from professional experience in that it is the domain of the collective environment and not the individual [4,5]. While research evidence is often treated as the most heavily weighted form, the PARIHS framers emphasize that all four forms have meaning and constitute evidence from the perspective of users.

**Context **comprises three components: (1) organizational culture, (2) leadership, and (3) evaluation [3,5]. Culture refers to the values, beliefs, and attitudes shared by members of the organization, and can emerge at the macro-organizational level, as well as among sub-units within the organization. Leadership includes elements of teamwork, control, decision making, effectiveness of organizational structures, and issues related to empowerment. Evaluation relates to how the organization measures its performance, and how (or whether) feedback is provided to people within the organization, as well as the quality of measurement and feedback.

**Facilitation **is defined as a "technique by which one person makes things easier for others" which is achieved through "support to help people change their attitudes, habits, skills, ways of thinking, and working" [1]. Facilitation is a human activity, enacted through roles. Its function is to help individuals and teams understand what they need to change and how to go about it [2,10]. That role may encompass a range of conventional activities and interventions, such as education, feedback and marketing [10], though two factors appear to distinguish facilitation, as defined in PARIHS, from other multifaceted interventions. First, as its name implies, facilitation emphasizes enabling (as opposed to doing for others) through critical reflection, empathy, and counsel. Second, facilitation is expressly responsive and interactive, whereas conventional multi-faceted interventions do not necessarily involve two-way communication. Stetler and colleagues provide a pithy illustration from an interview[10]:

On the site visit, I came in with a PowerPoint presentation. That is education. When they called me for help ... that was different. It was facilitation.

Harvey and colleagues propose that facilitation is an appointed role, as opposed to an opinion leader who is defined by virtue of his or her standing among peers [2]. Prior publications have also distinguished facilitation roles filled by individuals internal versus external to the team or organization implementing the evidenced-based practice [2,10]. Internal facilitators are local to the implementation team or organization, and are directly involved in the implementation, usually in an assigned role. They can serve as a major point of interface with external facilitators [10].

This distinction between internal and external facilitation may be particularly important in the context of assessing organizational readiness to change. Most prior publications on the PARIHS framework focused on external, rather than internal facilitation. (Stetler and colleagues even make the point of referring to internal facilitators by another name entirely: internal change agents [10]). However, for the purposes of assessing organizational readiness to change, internal facilitation may be most pertinent, because it is a function of the organization, and is therefore a constant whereas the external facilitation can be designed or developed according to the needs of the organization. Assessing the organization or team's initial state becomes the first step in external facilitation, guiding subsequent facilitation activities. This notion is consistent with the recent suggestion by researchers that PARIHS be used in a two-stage process, to assess evidence and context in order to design facilitation interventions [5].

The framers of PARIHS propose that the three core elements of evidence, context and facilitation have a cumulative effect [6]. They suggested that no element be presumed inherently more important than the others until empirically demonstrated so [1], and recently reiterated that relative weighting of elements and sub-elements is a key question that remains to be answered [5].

Developing a diagnostic and evaluative tool based on PARIHS is a priority for researchers who developed the framework [5]. Currently there are two published instruments based on PARIHS, both with important limitations.

The first is a survey to measure factors contributing to implementation of evidence-based clinical practice guidelines [13]. The survey was developed by researchers in Sweden and comprises 23 items addressing clinical experience, patient's experience, and clinical context. The latter includes items about culture, leadership, evaluation and facilitation. At the present time, only test-retest measurement reliability has been assessed, though with generally favorable results (Kappa scores ranging from 0.39 to 0.80). However, the English translation of the survey hews closely to the language used in the conceptual articles on PARIHS, and the authors report that respondents had difficulty understanding some questions. Specifically, questions about facilitation and facilitators were confusing for some respondents. In addition, the survey omits measures of research evidence and combines measures of facilitation as part of context. The survey has not been validated beyond test-retest reliability.

The second instrument, the Context Assessment Index, is a 37-item survey to assess the readiness of a clinical practice for research utilization or implementation [17]. The CAI scales were derived inductively from a multi-phase project combining expert panel input and exploratory factor analysis. The CAI comprises 5 scales: collaborative practice; evidence-informed practice; respect for persons; practice boundaries; and evaluation. It has been assessed using a sample of nurses from the Republic of Ireland and Northern Ireland, and found to have good internal consistency and test-retest reliability. However, the CAI measures general readiness for research utilization, rather than readiness for implementation of a specific, discrete practice change; the CAI is exclusively a measure of context, and does not assess perceptions of the evidence for a practice change. Also, although the items were based on PARIHS, the 5 scales were inductively derived and do not correspond with the conceptual sub-elements elaborated in the PARIHS writings. It is not clear what this means for the CAI as a measure of PARIHS elements.

### The organizational readiness to change assessment (ORCA)

A survey instrument [see Additional file 1] was developed by researchers from the Veterans Affairs Ischemic Heart Disease Quality Enhancement Research Initiative [18] for use in quality improvement projects as a tool for gauging overall site readiness and identifying specific barriers or challenges. The instrument grew out of the VA Key Players Study [19], which was a post-hoc implementation assessment of the Lipid Measurement and Management System study [20]. Interviews were conducted with staff at six study hospitals, each implementing different interventions, or sets of interventions, to improve lipid monitoring and treatment. The interviews revealed a number of common factors that facilitated or inhibited implementation, notably 1) communication among services; 2) physician prerogative in clinical care decisions; 3) initial planning for the intervention; 4) progress feedback; 5) specifying overall goals and evaluation of the intervention; 6) clarity of implementation team roles, 7) management support; and 8) resource availability.

IHD-QUERI investigators also referred to two other organizational surveys to identify major domains related to organizational change: 1) the Quality Improvement Implementation survey [21,22], a survey used to assess implementation of continuous quality improvement/total quality management in hospitals, and 2) the Service Line Research Project survey, which was used to assess implementation of service lines in hospitals [23]. The former comprises 7 scales: leadership; customer satisfaction; quality management; information and analysis; quality results; employee quality training; employee quality and planning involvement. The latter includes six scales: satisfaction, information, outlook, culture for change, teamwork, and professional development.

The ORCA survey comprises three major scales corresponding to the core elements of the PARIHS framework: (1) Strength and extent of evidence for the clinical practice changes represented by the QI program, assessed with four subscales, (2) Quality of the organizational context for the QI program, assessed with six subscales, and (3) Capacity for internal facilitation of the QI program, assessed with nine subscales. Each subscale comprised between three and six items assessing a common dimension of the given scale. Below, we briefly introduce and describe each of the 19 subscales.

#### Evidence

The evidence scale comprised four subscales. The first scale consists of two items that are meant to measure discord within the practice team about the evidence, that is, the extent to which the respondent sees his or her colleagues concluding a weaker or stronger evidence base than the respondent. The other three subscales correspond to the three hypothesized components of evidence in the PARIHS framework: research evidence, clinical experience and patient preferences.

The instrument omits items measuring the fourth hypothesized component of evidence, that of "routine information." Routine information did not appear in the original model [1], but was added in a 2004 update [8], after the ORCA was developed.

#### Context

Context comprises six subscales. Two subscales assess dimensions of organizational culture: one for senior leadership or clinical management, and one for staff members. Two subscales assess leadership practice: one focused on formal leadership, particularly in terms of teambuilding, and one focused on attitudes of opinion leaders for practice change in general (as a measure of informal leadership practice). One subscale assesses evaluation in terms of setting goals, and tracking and communicating performance. Context items are assessed relative to change or quality of care generally, and not relative to the specific change being implemented. For example, one item refers to opinion leaders and whether they believe that the current practice patterns can be improved; this does not necessarily mean they believe the specific change being implemented can improve current practice. This is important for understanding whether barriers to implementation relate to the specific change being proposed or to changing clinical processes more generally. Measuring readiness as a function of both the specific change and general readiness is an approach used successfully in models of organizational readiness to change outside of health care [24].

In addition, the ORCA includes a subscale measuring resources to support practice changes in general, once they had been made an organizational priority. General resources were added because research on organizational innovation suggests that slack resources, such as funds, staff time, facilities and equipment, are important determinants of successful implementation [25]. Later publications on PARIHS include resources, such as human, technology, equipment and financial as part of a receptive context for implementation [5].

#### Facilitation

Facilitation comprises nine elements focused on the organization's capacity for internal facilitation: (1) senior leadership management characteristics, such as proposing feasible projects and providing clear goals; (2) clinical champion characteristics, such as assuming responsibility for the success of the project and having authority to carry it out; (3) senior leadership or opinion leader roles, such as being informed and involved in implementation and agreeing on adequate resources to accomplish it; (4) implementation team member roles, such as having clearly defined responsibilities within the team and having release time to work on implementation; (5) implementation plan, such as having explicitly delineated roles and responsibilities, and obtaining staff input and opinions; (6) communication, such as having regular meetings with the implementation team, and providing feedback on implementation progress to clinical managers; (7) implementation progress, such as collecting feedback from patients and staff; (8) implementation resources, such as adequate equipment and materials, and incentives; and (9) implementation evaluation, such as staff and/or patient satisfaction, and review of findings by clinical leadership.

## Methods

We conducted two sets of psychometric analyses on cross-sectional, secondary data from three quality improvement projects conducted in the Veterans Health Administration.

### Data and Setting

Data came from surveys completed by staff participating in three quality improvement (QI) projects conducted between 2002 and 2006: 1) the Cardiac Care Initiative; 2) the Lipids Clinical Reminders project [26]; and 3) an intensive care unit quality improvement project. In each project, identical 77-item ORCA surveys were administered to one or more staff from each facility involved in quality improvement efforts. Respondents were asked to address issues related to that specific project. Each item measures the extent to which a respondent agrees or disagrees with the item statement on a 5-point Likert-type scale (1 = strongly disagree; 5 = strongly agree).

This study was reviewed and approved by the Institutional Review Boards at the University of Washington.

### Analyses

We conducted two sets of psychometric analyses: (1) item analysis to determine if items within scales correlate as predicted [27] and (2) exploratory factor analyses of the aggregated subscales to determine how many underlying "factors" might be present, and their relationships to each other [28].

The item analysis consisted of two measures of item correlation within a given subscale: (1) Cronbach's alpha was calculated for reliability; and (2) item-rest correlations were calculated as an indicator of convergent validity and to identify items that do not correlate well with a given subscale and could be dropped for parsimony. We used a minimum threshold of 0.80 for Cronbach's alpha [27], and assessed how dropping an item from its given subscale would affect the Cronbach's alpha for the subscale. We considered the minimum threshold 0.20 for item-rest correlation [27]. We also calculated Cronbach's alpha for the overall scales (e.g., evidence) as a function of the constituent subscales.

We conducted principal factors analysis with promax rotation to examine the emergent factor structure of the subscales and scales, and to determine if the data supported alternative factor solutions other than the three core elements hypothesized by the PARIHS framework. Following recommended procedures for latent variable analysis [29,30], we first separately factor analyzed the items comprising individual subscales to determine if the factor structure of the subscales was supported. We then factor analyzed the aggregated subscales.

We chose principal factors because it is commonly used for exploratory factor analysis and generally produces lower (and therefore more conservative) factor loadings than principal components analysis. We chose oblique rotation to allow the factors to correlate [31]. This is consistent with both the conceptual underpinnings of the framework which supposes that core elements are interrelated (e.g., facilitation may be influenced by context), and with the items used to operationalize the framework, which include common themes across scales (e.g., leadership culture and leadership implementation role).

We retained factors with (1) eigenvalues > = 1.0; (2) eigenvalues greater than the point at which the slope of decreasing eigenvalues approaches zero on a scree plot; and (3) two or more items loaded > = 0.60 [31]. We only retained factors that met all three criteria. Conversely, we eliminated subscales that failed to load on any factor at > = 0.40 for the individual subscales, and > = 0.60 for the aggregated subscales. A general rule of thumb is that the minimum sample for factor analysis is 10 observations per item, usually using a factor loading threshold of 0.40; the factor analyses of the individual subscales met this minimum sample size (as subscales comprise between 3 and 6 items), but not the factor analysis of the aggregated subscales (19 subscales). Methodological studies suggest that using higher factor loadings, such as 0.50 or 0.60, allows for stable factor solutions to be derived from much smaller samples [31]. Data were analyzed using STATA version 9.2.

## Results

### Descriptive Statistics

A total of 113 observations were available from the three QI projects: 1) the Cardiac Care Initiative (n = 65 from 49 facilities); 2) the Lipids Clinical Reminders project (n = 12 from 1 facility); and 3) the intensive care unit project (n = 36 from 9 facilities). Of these, 80 observations from 49 facilities were complete cases with no missing values: 1) the Cardiac Care Initiative (n = 48 from 42 facilities); 2) the Lipids Clinical Reminders project (n = 12 from 1 facility); and 3) the intensive care unit project (n = 20 from 8 facilities). For 105 of the 113 observations (93% of the sample), values were missing for fewer than 10 items, and for any given item, the number of observations missing values ranged from 1 to 8 (i.e., no item was missing for more than 8 of the 113 observations). Items were more likely to be missing later in the survey, suggesting potential respondent fatigue. Tables of missing values are available [see Additional file 2]. Findings below are based on the complete cases.

Mean scores on the subscales ranged from 2.25 (general resources subscale in the Lipids Reminders project sample) to 4.19 (research evidence subscale in the Lipids Reminders project sample) on a 5-point scale (Table 1). Across the three samples, clinical experience favoring the evidence-based practice changes was rated marginally lower, on average, than was the perceived research evidence, and the evidence in terms of patient preferences was rated lowest of the three evidence subscales. Among the subscales measuring context, staff culture was the highest rated in the Lipids Reminders and Cardiac Care Initiative projects, and opinion leaders was highest in the ICU QI Intervention. Across the three samples, the general resources subscale was the lowest rated of all subscales. Among the subscales measuring facilitation, leaders' practices was rated highest in the Lipids Reminders and Cardiac Care Initiative projects, and implementation plan was highest in the ICU QI Intervention. Across the three samples, the project resources subscale was the lowest rated of the facilitation subscales.

**Table 1:** Descriptive Statistics and Reliability for Organizational Readiness to Change Assessment Subscales

Scale and subscale labels (item numbers)	Number of items retained	Lipids-reminders (n = 12)		ICU QI-intervention (n = 20)		Cardiac Care Initiative (n = 48)		Overall (n = 80)		
		Mean	SD	Mean	SD	Mean	SD	Mean	SD	Cronbach's Alpha
Evidence Scale^†^	10	3.96	0.24	3.89	0.42	4.03	0.44	3.99	0.41	0.74^††^
Research (q3a – d)	3	4.19	0.48	4.08	0.57	4.18	0.47	4.16	0.49	0.68
Clinical experience (q4a – c)	3	4.08	0.35	3.98	0.58	4.15	0.54	4.10	0.52	0.77
Patient preferences (q5a – d)	4	3.60	0.43	3.61	0.45	3.77	0.53	3.71	0.49	0.68
Context Scale^†^	23	3.24	0.44	3.54	0.35	3.85	0.66	3.68	0.61	0.85^‡^
Leader culture (q6a – c)	3	2.92	0.93	3.50	0.83	3.91	0.98	3.66	1.00	0.92
Staff culture (q7a – d)	4	4.00	0.60	3.78	0.38	4.15	0.77	4.03	0.68	0.90
Leadership behavior (q8a – d)	4	2.92	0.90	3.61	0.47	3.95	0.90	3.71	0.88	0.93
Measurement (feedback) (q9a – d)	4	3.63	0.73	3.51	0.50	4.07	0.78	3.87	0.75	0.88
Opinion leaders (q10a – d)	4	3.73	0.38	3.85	0.49	4.10	0.68	3.98	0.61	0.91
General resources (q11a – d)	4	2.25	0.61	2.96	0.84	2.91	0.80	2.83	0.82	0.86
Facilitation Scale^†^	40	3.14	0.50	3.83	0.33	3.59	0.68	3.58	0.62	0.95
Leaders practices (q12a – d)	4	3.42	0.64	3.59	0.37	3.82	0.74	3.70	0.66	0.87
Clinical champion (q13a – d)	4	3.19	0.67	3.78	0.57	3.74	0.85	3.67	0.78	0.94
Leadership implementation roles (q14a – d)	4	2.94	0.68	3.85	0.50	3.73	0.67	3.64	0.70	0.87
Implementation team roles (q15a – d)	4	2.92	0.71	3.66	0.62	3.42	0.82	3.40	0.78	0.86
Implementation plan (q16a – d)	4	3.17	0.77	4.06	0.44	3.75	0.82	3.74	0.78	0.95
Project communication (q17a – d)	4	3.25	0.65	4.05	0.46	3.66	0.87	3.70	0.79	0.92
Project progress tracking (q18a – d)	4	3.25	0.51	3.94	0.49	3.44	0.70	3.53	0.67	0.82
Project resources and context (q19a – f)	6	2.86	0.63	3.53	0.47	3.27	0.77	3.27	0.71	0.87
Project evaluation (q20a – e)	5	3.30	0.67	4.04	0.40	3.49	0.67	3.60	0.66	0.87

† The three major scales (evidence, context, facilitation) are averages of their constituent subscales, thus subscales are equally weighted. ‡ Cronbach's alpha for a revised context scale after eliminating the general resources subscale was 0.87. †† Cronbach's alpha for a revised evidence scale based on just the research evidence and clinical experience subscales was 0.83. Alpha numeric information in parentheses is item numbers, which are used in the example survey [see Additional file 1].

### Item Analysis

Cronbach's alpha for scale reliability for the overall scales were 0.74, 0.85 and 0.95 for the evidence, context and facilitation scales, respectively. Cronbach's alpha for the constituent subscales ranged from 0.68 for the research evidence and patient experience subscales of the evidence scale to 0.95 for the implementation plan subscale of the facilitation scale (Table 1).

Three subscales, the three comprising the evidence scale, failed to meet the conventional threshold of 0.80 for reliability [27]. Cronbach's alphas were initially 0.44, 0.62 and to 0.70, for the research evidence, clinical experience and patient preference subscales, respectively. One item from the research evidence subscale, q3e (the practice change will fail to improve patient outcomes [see Additional file 1]), had an item-rest correlation of 0.10, failing to meet the threshold of 0.20. Eliminating this item improved the Cronbach's alpha to 0.54, but the item-rest correlation for item q3d (the practice change will improve patient outcomes, even though it is experimental) fell to 0.16. Dropping q3d further improved the Cronbach's alpha for the research evidence subscale to 0.68.

For the clinical experience subscale, item q4d (the practice change has not been previously attempted in the facility) had the lowest item-rest correlation at 0.25. Although it met the minimum threshold for item-rest correlations, the Cronbach's alpha for the subscale improved from 0.63 to 0.77 when item q4d was dropped from the subscale. These three items (q3e, q3d, and q4d) were excluded in subsequent analyses. This decision was based both on the reliability results, and because of the items appeared to address potentially distinct concepts, such as predicting the effect of the practice change on patient outcomes (this is further explained in the Discussion). The figures in Table 1 were calculated without these three items.

The patient preferences subscale failed to meet the 0.80 threshold for reliability, but item-rest correlations for all four items ranged from 0.42 to 0.50, well above the minimum threshold of 0.20. Eliminating any item decreased the Cronbach's alpha for the subscale. Although the subscales comprising the evidence scale failed to meet the minimum threshold for reliability, we elected to retain them for the factor analysis because of the high item-rest correlations and because the scale represented concepts central to the PARIHS model.

### Factor Analysis

First we factor analyzed the constituent items for each subscale. Based on the three criteria discussed in the methods section, all 19 factor analyses of the constituent items of the individual subscales produced single factor solutions. All item factor loadings exceeded the minimum threshold of 0.40, ranging from 0.45 for q3c in the research evidence subscale to 0.95 for q13d of the clinical champion subscale. Individual subscale factor analyses results are available [see Additional file 3] but not reported in the text.

Next we factor analyzed the aggregated subscales. Based on the three criteria discussed in the methods section, three factors were retained (Table 2). Based on the criterion of factor loading > = 0.60, seven of the nine facilitation subscales loaded onto the first factor; five of the six context subscales loaded onto the second factor; and the three evidence subscales loaded on the third factor. No subscales cross-loaded on multiple factors, and all subscales, except the leaders' practices subscale from the facilitation scale, loaded primarily on factors corresponding to the core element they were intended to measure. The subscale measuring leader practices had a factor loading of 0.76 on the second factor, which the majority of the context subscales loaded on.

**Table 2:** Exploratory factor analysis of Organizational Readiness to Change Assessment subscales (n = 80)

Retained Factors	Eigen-value	Proportion		
Factor1	7.61	0.59		
Factor2	7.12	0.55		
Factor3	3.23	0.25		
				
Principal factors with promax rotation	Factor 1	Factor 2	Factor 3	Uniqueness
Evidence Scale				
Research	-0.10	0.11	**0.74**	0.42
Clinical experience	0.04	0.01	**0.83**	0.27
Patient preferences	0.06	-0.24	**0.62**	0.67^†^
				
Context Scale				
Leader culture	0.07	**0.83**	-0.08	0.29
Staff culture	-0.17	**0.67**	0.26	0.48
Leadership behavior	0.08	**0.88**	-0.05	0.18
Measurement (leadership feedback)	0.07	**0.72**	0.01	0.41
Opinion leaders	0.04	**0.69**	0.12	0.41
General resources	0.41	0.10	0.13	0.71^†^
				
Facilitation Scale				
Leaders practices	0.24	**0.74**	-0.02	0.19
Clinical champion	0.49	0.35	0.15	0.34
Leadership implementation roles	**0.65**	0.33	-0.08	0.28
Implementation team roles	**0.67**	0.23	0.02	0.30
Implementation plan	**0.73**	0.34	-0.10	0.13
Project communication	**0.80**	0.12	0.07	0.20
Project progress tracking	**0.92**	-0.09	-0.02	0.25
Project resources and context	**0.86**	0.01	0.00	0.24
Project evaluation	**0.88**	-0.14	0.02	0.34

Factor loadings > = 0.60, our threshold, are bolded

† Indicates subscale for which factors failed account for > = 50% of variance.

General resources, from the context scale, and clinical champion role, from the facilitation scale, failed to load significantly on any of the factors, although both loaded primarily on the first factor, with the majority of facilitation subscales. The factor loadings were 0.41 and 0.49, respectively.

The uniqueness statistic for the general resources subscale of the context scale and the patient preference subscale of the evidence scale were 0.70 and 0.67, respectively. This suggests that the majority of variances in the two subscales were not accounted for by the three emerging factors taken together.

## Discussion

We find some statistical support, in terms of reliability and factor analyses, for aggregation of survey items and subscales into three scales of organizational readiness-to-change based on the core elements of the PARIHS framework: evidence, context and facilitation. Reliability statistics met conventional thresholds for the majority of subscales, indicating that the subscales intended to measure the individual components of the main elements of the framework (e.g., the six components of the context scale) held together reasonably well. Exploratory factor analysis applied to the aggregated subscale scores supports three underlying factors, with the majority of subscale scores clustered corresponding to the core elements of the PARIHS framework.

However, three findings may indicate concerns and suggest need for further revision to the instrument and further research on its reliability and validity: (1) reliability was poor for the three evidence subscales; (2) the subscales measuring clinical champion (as part of the facilitation scale), and availability of general resources (as part of the context scale) failed to load significantly on any factor; and (3) the leadership practices subscale loaded on the second factor with most of the context subscales. We discuss each of these in turn.

### Reliability of evidence subscales

Reliability, as measured by Cronbach's alpha, was mediocre for the evidence scale and the three constituent subscales. Poor reliability could be a function of too few items (alpha coefficients are highly sensitive to the number of items in a scale [27]); could indicate that the items are deficient measures of the evidence construct; or could signal that the subscales are not uni-dimensional, i.e., they reflect multiple underlying factors with none measured reliably or well.

There is some evidence for the latter given the observed improvement in reliability statistics after dropping three items: q3d and q3e from the research evidence subscale, and q4d from the practice experience subscale. These items had some important conceptual differences from other items in their respective subscales. Both q3d and q3e are about anticipating the effect of the practice change on patient outcomes, whereas the other items in the subscale (q3a – q3c) are about the scientific evidence for the practice change. The former require respondents to make a prediction about a future state, not just an assessment of a current one (i.e., the state of the research evidence). Item q4d, on the other hand, is about whether the practice change has previously been attempted in the respondent's clinical setting, which was unlikely given the context was quality improvement projects introducing new practices. However, factor analysis generally supported a common factor solution for the three subscales, supporting the hypothesis that the subscales may tap into a common latent variable. This question would benefit from more conceptual as well as empirical work.

The patient preferences subscale requires further consideration, and we feel remains an open question as to how it fits with the model and with the survey. It had high uniqueness, indicating that the majority of variance in the items was not accounted for by the three factors. Furthermore, past research appears to conflict with the contention that patient preferences or experiences have significant influence on how favorably clinicians evaluate a given practice or course of treatment. For example, some research concludes there is little or no correlation between patient preferences and what clinicians do [32,33], and even after interventions to increase shared decision making (a practice intended to better incorporate patient preferences into health care practice), the actual effects on clinical choices appear limited, even though providers and patients may perceive greater participation [34]. Patient preference *should *be a major driver of implementation of evidence-based practices, but we suspect that in our current health care system it is generally not. It remains unclear what this means for assessing patient preferences as a distinct component of organizational readiness to change, but additional exploratory research would seem to be in order.

It is also important to note that Cronbach's alpha findings do not mean that the evidence scale is invalid. The item-level results from the item-rest correlations suggested the evidence subscales had strong reliability, and the subscale-level principal factors analysis suggested a common, latent factor structure. Other researchers have demonstrated that Cronbach's alpha is not a measure of uni-dimensionality; it is possible to obtain a high alpha coefficient from a multidimensional scale, i.e., from a scale representing multiple constructs, and conversely to obtain a low alpha coefficient from a uni-dimensional scale [35]. Overall, the scale reliability findings for the evidence scale primarily suggest caution in interpreting the aggregated scale and that further study is warranted.

As noted in the background, the ORCA omits a subscale for routine information, which was added to the framework beginning in 2004 [8], and that could affect reliability for the overall evidence scale. However, this omission would not account for the weak reliability of the other subscales. Moreover, conceptually, routine information would appear more congruent with the context element. Routine information addresses the existence and use of data gathering and reporting systems, which are a function of the place where the evidence-based practice or technology is being implemented rather than a characteristic of the evidence-based practice itself or how it is perceived by users. In contrast, the other evidence subscales are dimensions of the perceived strength of the evidence, e.g., the strength of the research evidence; how well the new practice fits with past clinical experience. The meaning of a routine information subscale, as a dimension for evaluating the strength of the evidence, requires further consideration.

### Two subscales with low factor loadings

Two subscales failed to load significantly on any of the three factors: One measured dimensions of facilitation related to the clinical champion role, the other measured dimensions of context related to the availability of general resources, such as facilities and staffing. There are at least two ways to interpret this finding, with different attendant implications.

First, the failure of the two subscales to load on any of the three factors may indicate that overall availability of resources and clinical champion roles are functions of unique factors, distinct from evidence, context and facilitation (at least as framed in this instrument). Empirically and conceptually, we believe this may be the case for the general resource availability, but not for the clinical champion role.

In the case of general resource availability, the subscale had high uniqueness, indicating that a majority of variance of the items was not accounted for by any of the three factors. Conceptually, this subscale was not part of the original PARIHS framework; it was added to the ORCA based on other organizational research supporting the powerful influence of resource availability as an initial state that often sets boundaries in planning and execution. Although this seems to fit logically within the domain of the context scale, general resources may be a function of factors at other levels. This is consistent with the observed subscale scores, which were lowest for the general resources subscale across the three study samples. General resource availability may be less a function of the organization (in this case individual VHA facilities), and more a function of the broader resource environment in the VHA, or in the US health care system generally. The period covered in these three quality improvement projects has been one of high demand on Veterans Health Administration services [36], and cost containment was (and continues to be) a major and pervasive issue in healthcare [37]. We still believe that resource availability is an important factor in the determination of organizational readiness to change. However, it may be distinct from the three factors hypothesized in the PARIHS model, appearing different from the other dimension of context. We propose that additional conceptual work is needed on this subscale and that more items are likely needed to reliably measure it.

Second, the distinctiveness of the two subscales may indicate measurement error. General resource availability and clinical champion role might be appropriately understood as distinct reflections of the favorability of the context in the organization. However, the items, and their component subscales, may simply be inaccurate measures of the latent variables, or the number of observations in this analysis may have been insufficient for a stable estimate of the factors. We believe the latter is the case for the clinical champion subscale, which had a relatively low uniqueness value (0.34), and relatively high factor loading (0.49). Although the factor loading did not meet the threshold (0.60), we set an unusually high threshold for this analysis because the relatively small number of observations needed to be balanced with high factor loadings in order to achieve stable estimates [31]. We expect that repeating the analysis with a larger sample will confirm that the clinical champion subscale loads onto the same factor as the other facilitation subscales.

### The leadership practices subscale loaded on the context factor

The subscale measuring leaders' practices (from the facilitation scale) loaded on the second factor with context subscales. The leaders' practice subscale addressed whether senior leaders or clinical managers propose an appropriate, feasible project; provide clear goals; establish a project schedule; and designate a clinical champion. The high loading on the second factor could indicate that the leaders' practices subscale is properly understood as part of context, or it could signal poor discriminant validity between the context and facilitation scales. However, in this case, we believe the overlap may be a function measurement error related to item wording. Two of the items refer to "a project," which put the respondent in mind of a generic change more consonant to the questions in the context scale, whereas many of the facilitation items in the subsequent subscales refer to "this project" or "the intervention" implying the specific implementation project named in the question stem from the opening of the survey.

We believe that this unintended discrepancy in the pattern of wording cued respondents to answer the leader practices questions in a different frame of mind, conceiving of them in terms of projects in general rather than their estimate of leadership practices in the project they were actively engaged upon. This will be a revision to explore in future use of the survey.

Another question readers should bear in mind is whether readiness to change is best understood as a formative scale or a reflective scale. Principal factors analysis assumes that the individual items are reflective of common, latent variables (or factors) that cause the item responses [38,39]; when a scale is reflective, it corresponds to a given latent variable. However, organizational readiness to change may be more aptly understood as a formative scale, meaning that the constituent pieces (items or subscales) are the determinants and the latent variable organizational readiness to change is the intermediate outcome [38]. In the former case, the constituent parts are necessarily correlated (see Howell et al 2007 for a comparison of the mathematical assumptions underlying formative and reflective scales). For example, a scale meant to measure native athletic ability should register high correlations among constituent components meant to assess speed, strength, and agility; i.e., the physiological factors that determine speed, are also thought to determine strength and agility, and therefore a person scoring "high" on one component should score relatively high on the others. Conversely, a scale meant to measure how good a baseball player is, might assess their throwing, fielding, and batting to create a composite score. Throwing, fielding and batting may often be related – being in part a function of native athletic ability – but they're also a function of specific training activities and experience, and skill developed in one does not parlay into skill in the others. Rigorous training in pitching will not make you a good batter. For the purposes of the present analyses, we assumed that the ORCA is a reflective scale; the factor analysis appears to support that conclusion. However, the domains covered are quite diverse, and it seems appropriate to further explore the question of whether organizational readiness to change should properly be understood as a formative or a reflective scale.

### Limitations

There are five major limitations to our work. First, this analysis does not address the validity of the instrument as a predictor of evidence-based clinical practice, or even as a correlate of theoretically relevant covariates, such as implementation activities. Our objective with the present analysis was confined to assessing correlations among items within respondents to determine if the items cluster into scales and subscales as predicted. Criterion validation using implementation and quality-of-care outcomes is the next phase of our work.

Second, this study relied on secondary data from quality improvement projects, which did not employ some standard practices for survey development intended to mitigate threats to internal validity. We note two specific examples. First, the items were organized according to the predicted scales and subscales, rather than being presented to respondents in a random order. Item ordering can influence item scoring, and introduces the danger that reliability statistics may be inflated because items were organized according to the predicted subscales. However, this is not an uncommon practice in health services research survey instruments. Second, two of the quality improvement projects (Cardiac Care Initiative, and the intensive care unit quality improvement project) entailed multiple evidence-based practice changes, each of which could conceivably elicit different responses in terms of evidence, context and facilitation. The surveys assessed these practice changes as a whole, and therefore may have introduced measurement error to the extent that respondents perceived evidence, context and facilitation differently for different components. However, the danger here is less significant than for the item ordering, as the measurement error would tend to inflate item variance within scales, and therefore bias results towards the null (i.e., toward an undifferentiated mass of items rather than distinct scales), which we did not observe.

Third, the survey instrument is somewhat long (77 items), and may need to be shorter to be most useful. Despite the length, we note that most respondents are able to complete the survey in about 15 minutes, and this instrument is shorter than organizational readiness instruments used in other sectors, such as business and IT [40]. Moreover, any item reduction needs to consider the threat to content validity posed by potentially failing to measure an essential content domain [41]. The research presented included only preliminary item reduction based on scale reliability. Although scale reliability statistics often serve as a basis for excluding items [27], we believe that item reduction is best done as a function of criterion validation, i.e., that items are retained as a function of how much variance they account for in some theoretically meaningful outcome, and content validity, i.e., consideration of the theoretical domains the instrument is purported to measure. We regard this as a priority for the next stage of research.

Fourth, the sample size was small (80) relative to the number of survey items (77). This led us to factor analyze the aggregated subscales rather than the constituent items. This assumed that the subscales were unidimensional. While Cronbach's alpha findings generally supported the reliability of the subscales, high average correlations can still occur among items that reflect multiple factors [35], and high reliability is no guarantee that the subscales were unidimensional. This limitation will be corrected with time when additional data become available and the analysis can be repeated with a larger sample.

Fifth, the ORCA was fielded a single time in each project, which leaves unanswered questions both about the proper timing of the assessment and how variable subscales and scales are over time. In terms of timing, in the Lipids Clinical Reminders project, and the intensive care unit quality improvement project the instrument was fielded before any work related to the specific change was undertaken. In the case of the Cardiac Care Initiative, some work had already begun at some sites. It is possible that administering the instrument at more than one time point might yield different factor structures.

Other limitations include questions of external validity, for example, in terms of the setting in the VHA and these particular evidence-based practices; and questions of internal validity, in terms of the sensitivity of the measures to changes in wording or format. These limitations are all important topics for future research on the instrument.

## Conclusion

We find general support for the reliability and factor structure of an organizational readiness to change assessment based on the PARIHS framework. We find some discrepant results, in terms of poor reliability among subscales intended to measure distinct dimensions of evidence, and factor analysis results for measures of general resources and clinical champion role that do not conform to the PARIHS framework.

The next critical step is to use outcomes from implementation activities for criterion validation. This should provide more information about which items and scales are the most promising candidates for a revised readiness to change instrument.

## Abbreviations

ORCA: Organizational Readiness to Change Assessment; PARIHS: Promoting Action on Research Implementation in Health Services; VHA: Veterans Health Administration

## Competing interests

The authors declare that they have no competing interests.

## Authors' contributions

CDH conceived of the study and framed the research design, carried out the analyses, interpreted findings, and drafted the manuscript. YFL collaborated on study design, advised on the analyses, interpreted findings, and helped draft the manuscript. NDS led the development of the ORCA, helped frame the study, interpreted findings, and helped draft the manuscript. AES was a co-developer of the ORCA, helped frame the study, collected data in two of the three QI projects, and advised on the analyses, interpreted findings and helped draft the manuscript. All authors read and approved the final manuscript.

## Supplementary Material

Additional file 1 **Annotated copy of the Organizational Readiness to Change Assessment (ORCA)**. This is an annotated copy of the Organizational Readiness to Change Assessment (ORCA). Click here for file

Additional file 2 **Tables of missing values**. This file contains two tables, one showing missing values by observation, and the other showing missing values by item. Click here for file

Additional file 3 **Results of item-level factor analyses for individual subscales**. This file contains data tables for the factor analysis of the constituent items for each subscale, which we did prior to factor analyzing the aggregated subscales. Click here for file
