# Supplementary material for: Organizational readiness to change assessment (ORCA): Development of an instrument based on the Promoting Action on Research in Health Services (PARIHS) framework
Source: Implement Sci. 2009 Jul 14;4:38. doi: 10.1186/1748-5908-4-38 (PMC2716295; doi:10.1186/1748-5908-4-38)
Supplement: Additional file 1 — Annotated copy of the Organizational Readiness to Change Assessment (ORCA). This is an annotated copy of the Organizational Readiness to Change Assessment (ORCA). [file 1748-5908-4-38-S1.pdf]

Organizational Readiness to Change Assessment

Name of Station: ____

I. Evidence Assessment

Finding: Practice goals in the VISN ## Cardiac Care Plan will improve outcomes for patients with acute coronary syndromes.

Comment [Helfrich1]: This statement identifies the evidence-based practice being evaluated.

q1 Based on your assessment of the evidence basis for this statement, please rate the strength of the evidence in your opinion, on a scale of 1 to 5 where 1 is very weak evidence and 5 is very strong evidence:

very weak	weak	neither weak nor strong	strong	very strong	Don't know / Not applicable
1	2	3	4	5	99

q2 Now, please rate the strength of the evidence basis for this statement based on how you think respected clinical experts in your institution feel about the strength of the evidence, on a 1 to 5 scale similar to the one above:

very weak	weak	neither weak nor strong	strong	very strong	Don't know / Not applicable
1	2	3	4	5	99

These items are intended to be differenced and assessed separately from the other evidence subscales.

For each of the following statements, please rate the strength of your agreement with the statement, from 1 (strongly disagree) to 5 (strongly agree)

q3 (Research) The proposed practice changes or guideline implementation:

	strongly disagree	disagree	neither agree nor disagree	agree	strongly agree	Don't know/ Not Applicable
a) are(is) supported by RCTs or other scientific evidence from the VA	1	2	3	4	5	99
b) are(is) supported by RCTs or other scientific evidence from other health care systems	1	2	3	4	5	99
c) should be effective, based on current scientific knowledge	1	2	3	4	5	99
~~d) are(is) experimental, but may improve patient outcomes~~	~~1~~	~~2~~	~~3~~	~~4~~	~~5~~	~~99~~
~~e) likely won't make much difference in patient outcomes~~	~~1~~	~~2~~	~~3~~	~~4~~	~~5~~	~~99~~

These items were eliminated following subscale reliability analyses.

q4 (Clinical Experience) The proposed practice changes or guideline implementation:

	strongly disagree	disagree	neither agree nor disagree	agree	strongly agree	Don't know/ Not Applicable
a) are supported by clinical experience with VA patients	1	2	3	4	5	99
b) are supported by clinical experience with patients in other health care systems	1	2	3	4	5	99
c) conform to the opinions of clinical experts in this setting	1	2	3	4	5	99
~~d) have not been attempted in this clinical setting~~	~~1~~	~~2~~	~~3~~	~~4~~	~~5~~	~~99~~

This item was eliminated following subscale reliability analyses.

q5 (Patient Preferences) The proposed practice changes or guideline implementation:

	strongly disagree	disagree	neither agree nor disagree	agree	strongly agree	Don't know/ Not Applicable
a) have been well-accepted by VA patients in a pilot study	1	2	3	4	5	99
b) are consistent with clinical practices that have been accepted by VA patients	1	2	3	4	5	99
c) take into consideration the needs and preferences of VA patients	1	2	3	4	5	99
d) appear to have more advantages than disadvantages for VA patients	1	2	3	4	5	99

The patient preferences subscale had mediocre reliability but good factor structure; it may need additional items to improve reliability, or may need to be considered separately from the other evidence subscales.

II. Context Assessment

For each of the following statements, please rate the strength of your agreement with the statement, from 1 (strongly disagree) to 5 (strongly agree).

q6 (Culture) Senior leadership/clinical management in your organization:

	strongly disagree	disagree	neither agree nor disagree	agree	strongly agree	Don't know/ Not Applicable
a) reward clinical innovation and creativity to improve patient care	1	2	3	4	5	99
b) solicit opinions of clinical staff regarding decisions about patient care	1	2	3	4	5	99
c) seek ways to improve patient education and increase patient participation in treatment	1	2	3	4	5	99

q7 (Culture) Staff members in your organization:

	strongly disagree	disagree	neither agree nor disagree	agree	strongly agree	Don't know/ Not Applicable
a) have a sense of personal responsibility for improving patient care and outcomes	1	2	3	4	5	99
b) cooperate to maintain and improve effectiveness of patient care	1	2	3	4	5	99
c) are willing to innovate and/or experiment to improve clinical procedures	1	2	3	4	5	99
d) are receptive to change in clinical processes	1	2	3	4	5	99

q8 (Leadership) Senior leadership/Clinical management in your organization:

	strongly disagree	disagree	neither agree nor disagree	agree	strongly agree	Don't know/ Not Applicable
a) provide effective management for continuous improvement of patient care	1	2	3	4	5	99
b) clearly define areas of responsibility and authority for clinical managers and staff	1	2	3	4	5	99
c) promote team building to solve clinical care problems	1	2	3	4	5	99
d) promote communication among clinical services and units	1	2	3	4	5	99

q9 (Measurement) Senior Leadership/clinical management in your organization:

	strongly disagree	disagree	Neither agree nor disagree	agree	strongly agree	Don't know/ Not Applicable
a) provide staff with information on VA performance measures and guidelines	1	2	3	4	5	99
b) establish clear goals for patient care processes and outcomes	1	2	3	4	5	99
c) provide staff members with feedback/data on effects of clinical decisions	1	2	3	4	5	99
d) hold staff members accountable for achieving results	1	2	3	4	5	99

q10 (Readiness for change) Opinion leaders in your organization:

	strongly disagree	disagree	neither agree nor disagree	agree	strongly agree	Don't know/ Not Applicable
a) believe that the current practice patterns can be improved	1	2	3	4	5	99
b) encourage and support changes in practice patterns to improve patient care	1	2	3	4	5	99
c) are willing to try new clinical protocols	1	2	3	4	5	99
d) work cooperatively with senior leadership/clinical management to make appropriate changes	1	2	3	4	5	99

The opinion leaders subscale had good reliability but did not load on any factor at the factor loading threshold of 0.60 we set for this analysis. However, we think that's due to the high bar we set. Uniqueness (the amount of variation in the subscale unexplained by the factors) was low, and the factor loading of 0.49, would meet most conventional thresholds.

q11 (Resources) In general in my organization, when there is agreement that change needs to happen:

	strongly disagree	disagree	neither agree nor disagree	agree	strongly agree	Don't know/ Not Applicable
a) we have the necessary support in terms of budget or financial resources	1	2	3	4	5	99
b) we have the necessary support in terms of training	1	2	3	4	5	99
c) we have the necessary support in terms of facilities	1	2	3	4	5	99
d) we have the necessary support in terms of staffing	1	2	3	4	5	99

In factor analyses, the general resources subscale did not load on factors with the other context subscales, or on any other factor. This may represent a distinct core element, that should be assessed independent of context.

III. Facilitation Assessment:

For each of the following statements, please rate the strength of your agreement with the statement, from 1 (strongly disagree) to 5 (strongly agree):

q12 (Characteristics) Senior leadership/clinical management will:

	strongly disagree	disagree	neither agree nor disagree	agree	strongly agree	Don't know/ Not Applicable
a) propose a project that is appropriate and feasible	1	2	3	4	5	99
b) provide clear goals for improvement in patient care	1	2	3	4	5	99
c) establish a project schedule and deliverables	1	2	3	4	5	99
d) designate a clinical champion(s) for the project	1	2	3	4	5	99

The wording for the senior leadership characteristics subscale needs to be revised to address a specific project rather than a generic one to be consonant with the other facilitation subscales.

q13 (Characteristics) The Project Clinical Champion:

	strongly disagree	disagree	neither agree nor disagree	agree	strongly agree	Don't know/ Not Applicable
a) accepts responsibility for the success of this project	1	2	3	4	5	99
b) has the authority to carry out the implementation	1	2	3	4	5	99
c) is considered a clinical opinion leader	1	2	3	4	5	99
d) works well with the intervention team and providers	1	2	3	4	5	99

q14 (Role) Senior Leadership/Clinical management/staff opinion leaders:	strongly disagree	disagree	neither agree nor disagree	agree	strongly agree	Don't know/ Not Applicable
a) agree on the goals for this intervention	1	2	3	4	5	99
b) will be informed and involved in the intervention	1	2	3	4	5	99
c) agree on adequate resources to accomplish the intervention	1	2	3	4	5	99
d) set a high priority on the success of the intervention	1	2	3	4	5	99

q15 (Role) The implementation team members:	strongly disagree	disagree	neither agree nor disagree	agree	strongly agree	Don't know/ Not Applicable
a) share responsibility for the success of this project	1	2	3	4	5	99
b) have clearly defined roles and responsibilities	1	2	3	4	5	99
c) have release time or can accomplish intervention tasks within their regular work load	1	2	3	4	5	99
d) have staff support and other resources required for the project	1	2	3	4	5	99

q16 (Style) The implementation plan for this intervention:	strongly disagree	disagree	neither agree nor disagree	agree	strongly agree	Don't know/ Not Applicable
a) identifies specific roles and responsibilities	1	2	3	4	5	99
b) clearly describes tasks and timelines	1	2	3	4	5	99
c) includes appropriate provider/patient education	1	2	3	4	5	99
d) acknowledges staff input and opinions	1	2	3	4	5	99

q17 (Style) Communication will be maintained through:	strongly disagree	disagree	neither agree nor disagree	agree	strongly agree	Don't know/ Not Applicable
a) regular project meetings with the project champion and team members	1	2	3	4	5	99
b) involvement of quality management staff in project planning and implementation	1	2	3	4	5	99
c) regular feedback to clinical management on progress of project activities and resource needs	1	2	3	4	5	99
d) regular feedback to clinicians on effects of practice changes on patient care/outcomes	1	2	3	4	5	99

q18 (Style) Progress of the project will be measured by:	strongly disagree	disagree	neither agree nor disagree	agree	strongly agree	Don't know/ Not Applicable
a) collecting feedback from patients regarding proposed/implemented changes	1	2	3	4	5	99
b) collecting feedback from staff regarding proposed/implemented changes	1	2	3	4	5	99
c) developing and distributing regular performance measures to clinical staff	1	2	3	4	5	99
d) providing a forum for presentation/discussion of results and implications for continued improvements	1	2	3	4	5	99

q19 (Resources) The following are available to make the selected plan work:	strongly disagree	disagree	neither agree nor disagree	agree	strongly agree	Don't know/ Not Applicable
a) staff incentives	1	2	3	4	5	99
b) equipment and materials	1	2	3	4	5	99
c) patient awareness/need	1	2	3	4	5	99
d) provider buy-in	1	2	3	4	5	99
e) intervention team	1	2	3	4	5	99
f) evaluation protocol	1	2	3	4	5	99

q20 (Evaluation) Plans for evaluation and improvement of this intervention include:	strongly disagree	disagree	neither agree nor disagree	agree	strongly agree	Don't know/ Not Applicable
a) periodic outcome measurement	1	2	3	4	5	99
b) staff participation/satisfaction survey	1	2	3	4	5	99
c) patient satisfaction survey	1	2	3	4	5	99
d) dissemination plan for performance measures	1	2	3	4	5	99
e) review of results by clinical leadership	1	2	3	4	5	99
