# Supplementary material for: Organizational readiness to change assessment (ORCA): Development of an instrument based on the Promoting Action on Research in Health Services (PARIHS) framework
Source: Implement Sci. 2009 Jul 14;4:38. doi: 10.1186/1748-5908-4-38 (PMC2716295; doi:10.1186/1748-5908-4-38)
Supplement: Additional file 3 — Results of item-level factor analyses for individual subscales. This file contains data tables for the factor analysis of the constituent items for each subscale, which we did prior to factor analyzing the aggregated subscales. [file 1748-5908-4-38-S3.doc]

---------------------------------------------------------------------------------

log: J:\IHD-QuERI\ACS Objective 4\RTC responses\RTC_validation\Complete c

> ase EFA_2009-05-17.txt

log type: text

opened on: 17 May 2009, 01:13:51

. *******************************************************

.

. *Item-level factor analysis on constituent items for each of the 19 individual

> subscales

.

. *May 16, 2007

.

. *******************************************************

.

.

.

. * Total observations

.

. count

80

.

.

.

. * Respondent cohorts

.

. tab survey

Survey cohort from one of three studies | Freq. Percent Cum.

----------------------------------------+-----------------------------------

Lipids-reminder study |1 | 12 15.00 15.00

ICU QI-intervention study |2 | 20 25.00 40.00

Cardiac Care Initiative implementation | 48 60.00 100.00

----------------------------------------+-----------------------------------

Total | 80 100.00

.

.

.

. *Some notes about the exploratory factor analysis:

.

. * We use principal factors (or common factors) because, according to Floyd and

> colleagues (1995:291), principal factors method is most appropriate for explora

> tory factor analysis. Principal components factor analysis (PCF) should result

> in factor loadings that are biased higher.

.

.

.

. ** In general, we are assuming the following thresholds to assess EFA findings:

.

. * 1) The number of factors will be determined based on two criteria:

.

. * (1) the number of eigenvalues greater than one (Guttman 1954) and

.

. * (2) Cattell's scree plot indicating the point at which the curve of decreasin

> g eigenvalues changes from a decelerating decline to a flat gradual slope (Catt

> ell 1978).

.

. * 2) Following convention, we adopt factor solutions derived from factor loadin

> gs of 0.40 or greater; this was raised to 0.60 for the factor analysis of aggre

> gated subscales because the of the relatively large number of items (19 subscal

> es) relative to observations (80 complete case observations).

.

.

.

. ****************************

.

. *** I. Evidence scale and subscales (q1 - q5d)

.

. ****************************

.

. * The Evidence scale comprises items q1 to q5d, organized a priori into four su

> bscales: 1) generic evidence, 2) research, 3) clinical experience and 4) patien

> t preferences.

.

.

.

. *1. Generic evidence subscale item are meant to contrast the respondent's overa

> ll assessment of the strength of the evidence vs. their perception of their col

> leagues' overall assessment of the strength of the evidence; they are intended

> to be differenced and therefore conventional scale reliability and factor struc

> ture analyses are not appropriate.

.

.

.

.

.

. * 2. Research evidence subscale

.

. * Note that we have dropped two items (q3d & q3e) from this scale following sca

> le reliability testing.

.

. factor q3a q3b q3c , mineigen(1)

(obs=80)

Factor analysis/correlation Number of obs = 80

Method: principal factors Retained factors = 1

Rotation: (unrotated) Number of params = 3

--------------------------------------------------------------------------

Factor | Eigenvalue Difference Proportion Cumulative

-------------+------------------------------------------------------------

Factor1 | 1.25255 1.15324 1.1506 1.1506

Factor2 | 0.09931 0.36254 0.0912 1.2418

Factor3 | -0.26322 . -0.2418 1.0000

--------------------------------------------------------------------------

LR test: independent vs. saturated: chi2(3) = 55.46 Prob>chi2 = 0.0000

Factor loadings (pattern matrix) and unique variances

---------------------------------------

Variable | Factor1 | Uniqueness

-------------+----------+--------------

q3a | 0.6568 | 0.5686

q3b | 0.7893 | 0.3770

q3c | 0.4451 | 0.8019

---------------------------------------

.

.

.

. * 3. Clinical experience evidence subscale

.

. * Note that we have dropped one item (q4d) from this scale following scale reli

> ability testing.

.

. factor q4a q4b q4c , mineigen(1)

(obs=80)

Factor analysis/correlation Number of obs = 80

Method: principal factors Retained factors = 1

Rotation: (unrotated) Number of params = 3

--------------------------------------------------------------------------

Factor | Eigenvalue Difference Proportion Cumulative

-------------+------------------------------------------------------------

Factor1 | 1.42811 1.52081 1.2640 1.2640

Factor2 | -0.09269 0.11292 -0.0820 1.1820

Factor3 | -0.20561 . -0.1820 1.0000

--------------------------------------------------------------------------

LR test: independent vs. saturated: chi2(3) = 62.93 Prob>chi2 = 0.0000

Factor loadings (pattern matrix) and unique variances

---------------------------------------

Variable | Factor1 | Uniqueness

-------------+----------+--------------

q4a | 0.6915 | 0.5218

q4b | 0.7571 | 0.4268

q4c | 0.6138 | 0.6232

---------------------------------------

.

.

.

. * 4. Patient preference evidence subscale

.

. factor q5a q5b q5c q5d , mineigen(1)

(obs=80)

Factor analysis/correlation Number of obs = 80

Method: principal factors Retained factors = 1

Rotation: (unrotated) Number of params = 4

--------------------------------------------------------------------------

Factor | Eigenvalue Difference Proportion Cumulative

-------------+------------------------------------------------------------

Factor1 | 1.36428 1.12300 1.1518 1.1518

Factor2 | 0.24128 0.38188 0.2037 1.3555

Factor3 | -0.14060 0.13990 -0.1187 1.2368

Factor4 | -0.28050 . -0.2368 1.0000

--------------------------------------------------------------------------

LR test: independent vs. saturated: chi2(6) = 59.55 Prob>chi2 = 0.0000

Factor loadings (pattern matrix) and unique variances

---------------------------------------

Variable | Factor1 | Uniqueness

-------------+----------+--------------

q5a | 0.5697 | 0.6754

q5b | 0.6318 | 0.6008

q5c | 0.5672 | 0.6782

q5d | 0.5646 | 0.6813

---------------------------------------

.

.

.

. * Item analysis treating the aggregated Evidence "subscales" as items

.

. factor E1_divergent E2_research E3_experience E4_patients , mineigen(1)

(obs=77)

Factor analysis/correlation Number of obs = 77

Method: principal factors Retained factors = 1

Rotation: (unrotated) Number of params = 4

--------------------------------------------------------------------------

Factor | Eigenvalue Difference Proportion Cumulative

-------------+------------------------------------------------------------

Factor1 | 1.59945 1.27971 1.0152 1.0152

Factor2 | 0.31974 0.43644 0.2029 1.2181

Factor3 | -0.11670 0.11026 -0.0741 1.1441

Factor4 | -0.22696 . -0.1441 1.0000

--------------------------------------------------------------------------

LR test: independent vs. saturated: chi2(6) = 85.62 Prob>chi2 = 0.0000

Factor loadings (pattern matrix) and unique variances

---------------------------------------

Variable | Factor1 | Uniqueness

-------------+----------+--------------

E1_divergent | -0.2810 | 0.9210

E2_research | 0.8077 | 0.3476

E3_experie~e | 0.8016 | 0.3574

E4_patients | 0.4748 | 0.7746

---------------------------------------

.

.

.

. ****************************

.

. *** II. Context scale and subscales (q6a - q11d)

.

. ****************************

.

. * The Context scale comprises items q6a - q11d, organized a priori into six sub

> scales: 1) senior leadership / clinical manager culture, 2) staff culture, 3) s

> enior leadership / clinical management leadership, 4) use of measurement tools

> by leadership, 5) opinion leaders' readiness for change, and 6) availability of

> resources.

.

.

.

. *1. Senior leadership / clinical manager culture subscale

.

. factor q6a q6b q6c , mineigen(1)

(obs=80)

Factor analysis/correlation Number of obs = 80

Method: principal factors Retained factors = 1

Rotation: (unrotated) Number of params = 3

--------------------------------------------------------------------------

Factor | Eigenvalue Difference Proportion Cumulative

-------------+------------------------------------------------------------

Factor1 | 2.26837 2.35054 1.0880 1.0880

Factor2 | -0.08216 0.01909 -0.0394 1.0486

Factor3 | -0.10125 . -0.0486 1.0000

--------------------------------------------------------------------------

LR test: independent vs. saturated: chi2(3) = 168.63 Prob>chi2 = 0.0000

Factor loadings (pattern matrix) and unique variances

---------------------------------------

Variable | Factor1 | Uniqueness

-------------+----------+--------------

q6a | 0.8496 | 0.2781

q6b | 0.8875 | 0.2124

q6c | 0.8712 | 0.2411

---------------------------------------

.

.

.

. *2. Staff culture subscale

.

. factor q7a q7b q7c q7d , mineigen(1)

(obs=80)

Factor analysis/correlation Number of obs = 80

Method: principal factors Retained factors = 1

Rotation: (unrotated) Number of params = 4

--------------------------------------------------------------------------

Factor | Eigenvalue Difference Proportion Cumulative

-------------+------------------------------------------------------------

Factor1 | 2.87127 2.79597 1.0373 1.0373

Factor2 | 0.07530 0.12283 0.0272 1.0645

Factor3 | -0.04753 0.08343 -0.0172 1.0473

Factor4 | -0.13096 . -0.0473 1.0000

--------------------------------------------------------------------------

LR test: independent vs. saturated: chi2(6) = 237.39 Prob>chi2 = 0.0000

Factor loadings (pattern matrix) and unique variances

---------------------------------------

Variable | Factor1 | Uniqueness

-------------+----------+--------------

q7a | 0.8630 | 0.2552

q7b | 0.9210 | 0.1518

q7c | 0.8387 | 0.2966

q7d | 0.7582 | 0.4252

---------------------------------------

.

.

.

. *3. Senior leadership / clinical management leadership subscale

.

. factor q8a q8b q8c q8d , mineigen(1)

(obs=80)

Factor analysis/correlation Number of obs = 80

Method: principal factors Retained factors = 1

Rotation: (unrotated) Number of params = 4

--------------------------------------------------------------------------

Factor | Eigenvalue Difference Proportion Cumulative

-------------+------------------------------------------------------------

Factor1 | 3.05372 3.04269 1.0401 1.0401

Factor2 | 0.01102 0.01213 0.0038 1.0438

Factor3 | -0.00111 0.12652 -0.0004 1.0435

Factor4 | -0.12762 . -0.0435 1.0000

--------------------------------------------------------------------------

LR test: independent vs. saturated: chi2(6) = 266.12 Prob>chi2 = 0.0000

Factor loadings (pattern matrix) and unique variances

---------------------------------------

Variable | Factor1 | Uniqueness

-------------+----------+--------------

q8a | 0.8834 | 0.2196

q8b | 0.8467 | 0.2831

q8c | 0.8982 | 0.1933

q8d | 0.8659 | 0.2503

---------------------------------------

.

.

.

. *4. Use of measurement tools by leadership subscale

.

. factor q9a q9b q9c q9d , mineigen(1)

(obs=80)

Factor analysis/correlation Number of obs = 80

Method: principal factors Retained factors = 1

Rotation: (unrotated) Number of params = 4

--------------------------------------------------------------------------

Factor | Eigenvalue Difference Proportion Cumulative

-------------+------------------------------------------------------------

Factor1 | 2.66843 2.56733 1.0415 1.0415

Factor2 | 0.10110 0.13691 0.0395 1.0809

Factor3 | -0.03581 0.13572 -0.0140 1.0669

Factor4 | -0.17153 . -0.0669 1.0000

--------------------------------------------------------------------------

LR test: independent vs. saturated: chi2(6) = 198.42 Prob>chi2 = 0.0000

Factor loadings (pattern matrix) and unique variances

---------------------------------------

Variable | Factor1 | Uniqueness

-------------+----------+--------------

q9a | 0.8553 | 0.2685

q9b | 0.8536 | 0.2715

q9c | 0.8348 | 0.3031

q9d | 0.7151 | 0.4886

---------------------------------------

.

.

.

. *5. Opinion leaders' readiness for change subscale

.

. factor q10a q10b q10c q10d , mineigen(1)

(obs=80)

Factor analysis/correlation Number of obs = 80

Method: principal factors Retained factors = 1

Rotation: (unrotated) Number of params = 4

--------------------------------------------------------------------------

Factor | Eigenvalue Difference Proportion Cumulative

-------------+------------------------------------------------------------

Factor1 | 2.82417 2.74205 1.0554 1.0554

Factor2 | 0.08212 0.19123 0.0307 1.0861

Factor3 | -0.10911 0.01218 -0.0408 1.0453

Factor4 | -0.12128 . -0.0453 1.0000

--------------------------------------------------------------------------

LR test: independent vs. saturated: chi2(6) = 219.87 Prob>chi2 = 0.0000

Factor loadings (pattern matrix) and unique variances

---------------------------------------

Variable | Factor1 | Uniqueness

-------------+----------+--------------

q10a | 0.8230 | 0.3227

q10b | 0.8801 | 0.2255

q10c | 0.8297 | 0.3116

q10d | 0.8270 | 0.3160

---------------------------------------

.

.

.

. *6. Availability of resources subscale

.

. factor q11a q11b q11c q11d , mineigen(1)

(obs=80)

Factor analysis/correlation Number of obs = 80

Method: principal factors Retained factors = 1

Rotation: (unrotated) Number of params = 4

--------------------------------------------------------------------------

Factor | Eigenvalue Difference Proportion Cumulative

-------------+------------------------------------------------------------

Factor1 | 2.50315 2.28627 1.0117 1.0117

Factor2 | 0.21688 0.30502 0.0877 1.0993

Factor3 | -0.08813 0.06947 -0.0356 1.0637

Factor4 | -0.15760 . -0.0637 1.0000

--------------------------------------------------------------------------

LR test: independent vs. saturated: chi2(6) = 198.83 Prob>chi2 = 0.0000

Factor loadings (pattern matrix) and unique variances

---------------------------------------

Variable | Factor1 | Uniqueness

-------------+----------+--------------

q11a | 0.9147 | 0.1634

q11b | 0.5648 | 0.6810

q11c | 0.7535 | 0.4323

q11d | 0.8831 | 0.2202

---------------------------------------

.

.

.

. * Item analysis treating the six aggregated Context "subscales" as items

.

. factor C1_leader_cult C2_staff_cult C3_leadership C4_measurement C5_opinion_lea

> d C6_resources , mineigen(1)

(obs=80)

Factor analysis/correlation Number of obs = 80

Method: principal factors Retained factors = 1

Rotation: (unrotated) Number of params = 6

--------------------------------------------------------------------------

Factor | Eigenvalue Difference Proportion Cumulative

-------------+------------------------------------------------------------

Factor1 | 3.23251 2.97291 0.9698 0.9698

Factor2 | 0.25960 0.14964 0.0779 1.0477

Factor3 | 0.10996 0.04388 0.0330 1.0806

Factor4 | 0.06608 0.19648 0.0198 1.1005

Factor5 | -0.13040 0.07408 -0.0391 1.0613

Factor6 | -0.20448 . -0.0613 1.0000

--------------------------------------------------------------------------

LR test: independent vs. saturated: chi2(15) = 258.80 Prob>chi2 = 0.0000

Factor loadings (pattern matrix) and unique variances

---------------------------------------

Variable | Factor1 | Uniqueness

-------------+----------+--------------

C1_leader_~t | 0.8391 | 0.2960

C2_staff_c~t | 0.6680 | 0.5538

C3_leaders~p | 0.8942 | 0.2003

C4_measure~t | 0.7723 | 0.4036

C5_opinion~d | 0.7320 | 0.4641

C6_resources | 0.3877 | 0.8497

---------------------------------------

.

.

.

. ******************************

.

. *** III. Facilitation scale and subscales (q12a - q20e)

.

. ******************************

.

. * The Facilitation scale comprises items q12a - q20e, organized a priori into n

> ine subscales: 1) senior leadership / clinical management characteristics, 2) c

> linical champion characteristics, 3) senior leadership / clinical management ro

> les, 4) implementation team members' roles, 5) implementation plan, 6) communic

> ation style, 7) progress measurement, 8) resource availability, and 9) evaluati

> on plans.

.

.

.

. *1. Senior leadership / clinical management characteristics subscale

.

. factor q12a q12b q12c q12d , mineigen(1)

(obs=80)

Factor analysis/correlation Number of obs = 80

Method: principal factors Retained factors = 1

Rotation: (unrotated) Number of params = 4

--------------------------------------------------------------------------

Factor | Eigenvalue Difference Proportion Cumulative

-------------+------------------------------------------------------------

Factor1 | 2.58987 2.55221 1.0902 1.0902

Factor2 | 0.03766 0.16191 0.0159 1.1060

Factor3 | -0.12425 0.00333 -0.0523 1.0537

Factor4 | -0.12758 . -0.0537 1.0000

--------------------------------------------------------------------------

LR test: independent vs. saturated: chi2(6) = 179.95 Prob>chi2 = 0.0000

Factor loadings (pattern matrix) and unique variances

---------------------------------------

Variable | Factor1 | Uniqueness

-------------+----------+--------------

q12a | 0.8165 | 0.3334

q12b | 0.8584 | 0.2632

q12c | 0.8115 | 0.3414

q12d | 0.7266 | 0.4721

---------------------------------------

.

.

.

. *2. Clinical champion characteristics subscale

.

. factor q13a q13b q13c q13d , mineigen(1)

(obs=80)

Factor analysis/correlation Number of obs = 80

Method: principal factors Retained factors = 1

Rotation: (unrotated) Number of params = 4

--------------------------------------------------------------------------

Factor | Eigenvalue Difference Proportion Cumulative

-------------+------------------------------------------------------------

Factor1 | 3.16324 3.17513 1.0352 1.0352

Factor2 | -0.01190 0.01449 -0.0039 1.0313

Factor3 | -0.02639 0.04295 -0.0086 1.0227

Factor4 | -0.06933 . -0.0227 1.0000

--------------------------------------------------------------------------

LR test: independent vs. saturated: chi2(6) = 319.85 Prob>chi2 = 0.0000

Factor loadings (pattern matrix) and unique variances

---------------------------------------

Variable | Factor1 | Uniqueness

-------------+----------+--------------

q13a | 0.9086 | 0.1745

q13b | 0.7467 | 0.4425

q13c | 0.9349 | 0.1259

q13d | 0.9519 | 0.0939

---------------------------------------

.

.

.

. *3. Senior leadership / clinical management roles subscale

.

. factor q14a q14b q14c q14d , mineigen(1)

(obs=80)

Factor analysis/correlation Number of obs = 80

Method: principal factors Retained factors = 1

Rotation: (unrotated) Number of params = 4

--------------------------------------------------------------------------

Factor | Eigenvalue Difference Proportion Cumulative

-------------+------------------------------------------------------------

Factor1 | 2.48404 2.31566 1.0499 1.0499

Factor2 | 0.16838 0.28492 0.0712 1.1211

Factor3 | -0.11654 0.05344 -0.0493 1.0718

Factor4 | -0.16998 . -0.0718 1.0000

--------------------------------------------------------------------------

LR test: independent vs. saturated: chi2(6) = 170.88 Prob>chi2 = 0.0000

Factor loadings (pattern matrix) and unique variances

---------------------------------------

Variable | Factor1 | Uniqueness

-------------+----------+--------------

q14a | 0.6993 | 0.5110

q14b | 0.8443 | 0.2871

q14c | 0.7871 | 0.3805

q14d | 0.8140 | 0.3374

---------------------------------------

.

.

.

. *4. Implementation team members' roles subscale

.

. factor q15a q15b q15c q15d , mineigen(1)

(obs=80)

Factor analysis/correlation Number of obs = 80

Method: principal factors Retained factors = 1

Rotation: (unrotated) Number of params = 4

--------------------------------------------------------------------------

Factor | Eigenvalue Difference Proportion Cumulative

-------------+------------------------------------------------------------

Factor1 | 2.48043 2.02743 0.9244 0.9244

Factor2 | 0.45300 0.52487 0.1688 1.0932

Factor3 | -0.07187 0.10645 -0.0268 1.0665

Factor4 | -0.17832 . -0.0665 1.0000

--------------------------------------------------------------------------

LR test: independent vs. saturated: chi2(6) = 191.64 Prob>chi2 = 0.0000

Factor loadings (pattern matrix) and unique variances

---------------------------------------

Variable | Factor1 | Uniqueness

-------------+----------+--------------

q15a | 0.7770 | 0.3962

q15b | 0.8004 | 0.3594

q15c | 0.7835 | 0.3862

q15d | 0.7888 | 0.3777

---------------------------------------

.

.

.

. *5. Implementation plan subscale

.

. factor q16a q16b q16c q16d , mineigen(1)

(obs=80)

Factor analysis/correlation Number of obs = 80

Method: principal factors Retained factors = 1

Rotation: (unrotated) Number of params = 4

--------------------------------------------------------------------------

Factor | Eigenvalue Difference Proportion Cumulative

-------------+------------------------------------------------------------

Factor1 | 3.25743 3.20686 1.0279 1.0279

Factor2 | 0.05057 0.09375 0.0160 1.0439

Factor3 | -0.04318 0.05268 -0.0136 1.0302

Factor4 | -0.09585 . -0.0302 1.0000

--------------------------------------------------------------------------

LR test: independent vs. saturated: chi2(6) = 320.84 Prob>chi2 = 0.0000

Factor loadings (pattern matrix) and unique variances

---------------------------------------

Variable | Factor1 | Uniqueness

-------------+----------+--------------

q16a | 0.8973 | 0.1948

q16b | 0.8926 | 0.2033

q16c | 0.8979 | 0.1937

q16d | 0.9216 | 0.1507

---------------------------------------

.

.

.

. *6. Communication style subscale

.

. factor q17a q17b q17c q17d , mineigen(1)

(obs=80)

Factor analysis/correlation Number of obs = 80

Method: principal factors Retained factors = 1

Rotation: (unrotated) Number of params = 4

--------------------------------------------------------------------------

Factor | Eigenvalue Difference Proportion Cumulative

-------------+------------------------------------------------------------

Factor1 | 2.99168 2.90450 1.0269 1.0269

Factor2 | 0.08718 0.13873 0.0299 1.0568

Factor3 | -0.05156 0.06248 -0.0177 1.0391

Factor4 | -0.11403 . -0.0391 1.0000

--------------------------------------------------------------------------

LR test: independent vs. saturated: chi2(6) = 272.03 Prob>chi2 = 0.0000

Factor loadings (pattern matrix) and unique variances

---------------------------------------

Variable | Factor1 | Uniqueness

-------------+----------+--------------

q17a | 0.8402 | 0.2940

q17b | 0.7588 | 0.4243

q17c | 0.9205 | 0.1526

q17d | 0.9287 | 0.1374

---------------------------------------

.

.

.

. *7. Progress measurement subscale

.

. factor q18a q18b q18c q18d , mineigen(1)

(obs=80)

Factor analysis/correlation Number of obs = 80

Method: principal factors Retained factors = 1

Rotation: (unrotated) Number of params = 4

--------------------------------------------------------------------------

Factor | Eigenvalue Difference Proportion Cumulative

-------------+------------------------------------------------------------

Factor1 | 2.27198 2.03352 1.0252 1.0252

Factor2 | 0.23846 0.36079 0.1076 1.1328

Factor3 | -0.12233 0.04959 -0.0552 1.0776

Factor4 | -0.17192 . -0.0776 1.0000

--------------------------------------------------------------------------

LR test: independent vs. saturated: chi2(6) = 154.84 Prob>chi2 = 0.0000

Factor loadings (pattern matrix) and unique variances

---------------------------------------

Variable | Factor1 | Uniqueness

-------------+----------+--------------

q18a | 0.5444 | 0.7036

q18b | 0.8858 | 0.2153

q18c | 0.6956 | 0.5161

q18d | 0.8409 | 0.2929

---------------------------------------

.

.

.

. *8. Resource availability subscale

.

. factor q19a q19b q19c q19d q19e q19f , mineigen(1)

(obs=80)

Factor analysis/correlation Number of obs = 80

Method: principal factors Retained factors = 1

Rotation: (unrotated) Number of params = 6

--------------------------------------------------------------------------

Factor | Eigenvalue Difference Proportion Cumulative

-------------+------------------------------------------------------------

Factor1 | 3.25974 2.83420 0.9796 0.9796

Factor2 | 0.42554 0.37493 0.1279 1.1075

Factor3 | 0.05061 0.13112 0.0152 1.1227

Factor4 | -0.08051 0.06450 -0.0242 1.0985

Factor5 | -0.14500 0.03769 -0.0436 1.0549

Factor6 | -0.18269 . -0.0549 1.0000

--------------------------------------------------------------------------

LR test: independent vs. saturated: chi2(15) = 246.34 Prob>chi2 = 0.0000

Factor loadings (pattern matrix) and unique variances

---------------------------------------

Variable | Factor1 | Uniqueness

-------------+----------+--------------

q19a | 0.6524 | 0.5744

q19b | 0.6960 | 0.5155

q19c | 0.6583 | 0.5666

q19d | 0.7689 | 0.4088

q19e | 0.8197 | 0.3281

q19f | 0.8082 | 0.3468

---------------------------------------

.

.

.

. *9. Evaluation plans subscale

.

. factor q20a q20b q20c q20d q20e , mineigen(1)

(obs=80)

Factor analysis/correlation Number of obs = 80

Method: principal factors Retained factors = 1

Rotation: (unrotated) Number of params = 5

--------------------------------------------------------------------------

Factor | Eigenvalue Difference Proportion Cumulative

-------------+------------------------------------------------------------

Factor1 | 3.10990 2.23716 0.8211 0.8211

Factor2 | 0.87274 0.84655 0.2304 1.0515

Factor3 | 0.02619 0.10429 0.0069 1.0584

Factor4 | -0.07810 0.06505 -0.0206 1.0378

Factor5 | -0.14315 . -0.0378 1.0000

--------------------------------------------------------------------------

LR test: independent vs. saturated: chi2(10) = 333.43 Prob>chi2 = 0.0000

Factor loadings (pattern matrix) and unique variances

---------------------------------------

Variable | Factor1 | Uniqueness

-------------+----------+--------------

q20a | 0.8361 | 0.3009

q20b | 0.6843 | 0.5317

q20c | 0.6261 | 0.6080

q20d | 0.8629 | 0.2554

q20e | 0.8978 | 0.1940

---------------------------------------

.

.

.

. * Item analysis treating the nine aggregated Facilitation "subscales" as items

.

. factor F1_leadership F2_champion F3_lead_role F4_team_role F5_plan_style F6_com

> municat_style F7_progress_measure F8_resources F9_evaluation , mineigen(1)

(obs=80)

Factor analysis/correlation Number of obs = 80

Method: principal factors Retained factors = 1

Rotation: (unrotated) Number of params = 9

--------------------------------------------------------------------------

Factor | Eigenvalue Difference Proportion Cumulative

-------------+------------------------------------------------------------

Factor1 | 6.22185 5.74209 0.9590 0.9590

Factor2 | 0.47976 0.36399 0.0739 1.0329

Factor3 | 0.11577 0.11015 0.0178 1.0508

Factor4 | 0.00563 0.02793 0.0009 1.0517

Factor5 | -0.02230 0.02497 -0.0034 1.0482

Factor6 | -0.04727 0.00510 -0.0073 1.0409

Factor7 | -0.05237 0.04210 -0.0081 1.0329

Factor8 | -0.09447 0.02424 -0.0146 1.0183

Factor9 | -0.11871 . -0.0183 1.0000

--------------------------------------------------------------------------

LR test: independent vs. saturated: chi2(36) = 664.75 Prob>chi2 = 0.0000

Factor loadings (pattern matrix) and unique variances

---------------------------------------

Variable | Factor1 | Uniqueness

-------------+----------+--------------

F1_leaders~p | 0.7509 | 0.4361

F2_champion | 0.7772 | 0.3959

F3_lead_role | 0.8279 | 0.3146

F4_team_role | 0.8377 | 0.2983

F5_plan_st~e | 0.9259 | 0.1427

F6_communi~e | 0.8985 | 0.1926

F7_progres~e | 0.8213 | 0.3255

F8_resources | 0.8569 | 0.2658

F9_evaluat~n | 0.7703 | 0.4067

---------------------------------------
